# The prognostic effect of metastasis patterns on overall survival in organ metastatic lung adenocarcinoma

**DOI:** 10.1097/MD.0000000000033297

**Published:** 2022-04-07

**Authors:** Xiaowei Tie, Jin Wang, Yong Wang, Baobao Fu, Chenchen Wang, Xiaomin Li, Qianqian Jia, Fang Wang, Simeng Chen, Yanshun Zhang

**Affiliations:** a The First Affiliated Hospital of Anhui University of Science & Technology, HeFei, AnHui, China; b Bozhou People's Hospital, Bozhou, Qiaocheng, China

**Keywords:** lung adenocarcinoma, metastasis pattern, overall survival, SEER

## Abstract

The effect of various metastases patterns on the prognosis of lung adenocarcinoma (AD) remains unknown. The purpose of our retrospective study is to determine whether various metastases patterns have a prognostic impact on patients with organ metastatic lung AD. Patient data was extracted from the Surveillance, Epidemiology, and End Results (SEER) database. The Kaplan–Meier method was chosen for the evaluation of overall survival (OS) rate. Univariable and multivariable Cox regression analyses were conducted to evaluate independent prognostic factors. In the SEER database, 12,228 patients with IV lung AD were retrieved in total. And 78.78% of those patients (9633/12,228) suffered from one of brain, lung, liver or bone metastasis due to disease progression. It was found that the most common site in patients with metastatic lung AD was brain (21.20%), and the least common site of metastasis was liver (3.50%). Patients who suffered single lung metastatic showed relatively good OS, and the median survival time was 11 months (95% CI 0.470–0.516). For those with 2 metastatic sites, data analysis suggested that the median survival times of patients with bone and lung metastasis (10 months; 95% CI 0.469–0.542) were better than others. For those with 3 metastatic sites, data analysis suggested that metastatic pattern had no effect on the OS. Brain is the most common single metastasis site of lung AD. Compared with the other 3 metastatic sites, patients with lung metastasis had better survival results. Deeper knowledge of metastatic patterns will help doctors to better understand the prognosis and formulate more appropriate treatment plans.

## 1. Introduction

Lung cancer is one of the most common malignant tumor, with a 5-year survival rate below 16%.^[1–3]^ And non-small-cell lung carcinoma (NSCLC) and small-cell carcinoma lung cancer account for approximately 80% to 85% and 15% to 20%^[4]^ of it. NSCLC is generally divided into adenocarcinoma (AD) and squamous cell carcinoma. AD is the most common type, accounting for about 40% of NSCLC, while squamous cell carcinoma accounts for 25%.^[5]^ Tumor metastasis and recurrence remain challenging clinical issues. Surgery, chemoradiotherapy, targeted therapy, and immunotherapy are currently used to treat lung AD, but the prognosis remains dismal.^[6,7]^

The main cause of poor prognosis of lung AD is metastasis. Therefore, a better understanding of lung AD, especially the metastatic pattern of lung AD, may lead to more appropriate medical decisions and improved patient survival. Some clinical factors, such as gender, age, histological subtype and surgery, are related to survival of metastatic lung AD.^[8,9]^ However, these elements are in ignorance of the location and quantity of organ metastatic sites. Approximately 57% of lung AD have advanced to metastatic diseases, and the prognosis of patients is adversely affected by organ metastasis.^[10–12]^ Lung, bone, brain, pleura and liver are the common metastatic organs of LUAD.^[9]^ New predictive tools and devices are urgently needed in clinical practice for better prognosis of lung AD.

Patterns of metastasis have been found to be important prognostic implications in clinical work, so understanding the organ metastasis patterns can help us refine our assessment before treatment. By collating and analyzing data from the Surveillance, Epidemiology, and End Results database (SEER) database, we described organ metastatic sites and metastasis patterns. With more timely diagnosis and more alternative treatments, the survival time of lung AD is prolonged, and the quality of life is improved. Based on the development of imaging technology, the detection rate of micrometastases is increasing. Therefore, deeper knowledge of metastatic patterns will help doctors to better evaluate the prognosis of patients and formulate more appropriate treatment plans.

## 2. Methods

### 2.1. Patients

The SEER database is a free statistical database for the public, which collects a large amount of confirmatory medical data. All our data was downloaded from the SEER database. A total of 12,288 patients with metastatic lung AD were collected for data analysis. Eligible criteria were as follows: Patients diagnosed with metastatic lung AD (histologically confirmed) from 2010 to 2015. Lung AD was the first primary malignancy.

### 2.2. Statistical analysis

Pearson’ s Chi-square test was made use of comparing distribution frequency and clinicopathological characteristics. We applied the Kaplan–Meier method to assess the survival curve and performed the log-rank test to compare the OS. Multivariate Cox regression analysis was applied to screen out the relative prognostic factors. *P* < .05 was considered statistically significant. Statistical software SPSS Statistics 25.0.0 and R software (R version 4.1.0; https://cran.r-project.org/bin/windows/base/old/4.1.0/) were used for data analysis.

## 3. Results

### 3.1. Patient demographics

A total of 12,228 patients with IV lung AD were retrieved from the SEER database. Since the SEER database only provides patient data on bone, brain, liver, lung metastases, this study focused on patients with organ metastatic lung AD.

78.78% of the patients (9633/12,228) suffered from one of brain, lung, liver or bone metastasis due to disease progression.

### 3.2. Metastasis pattern

Table 1 presents the distribution of clinical characteristics of these patients. The distribution of race, sex and chemotherapy with or without bone metastasis had statistical significance (*P* < .05), but not among people with liver, lung, or brain metastases (*P* > .05). The distribution of age, tumor size, primary site, and surgery with and without bone or liver metastasis had statistical significance (*P* < .05), but not among patients with lung or brain metastases (*P* > .05). Just as shown in Table 1, there were significant differences, including grade staging and nodal stage (all, *P* < .001).

**Table 1:** Clinical features and metastasis sites.

Features	Bone metastasis		*P* value	Brain metastasis		*P* value	Liver metastasis		*P* value	Lung metastasis		*P* value
	Yes	No		Yes	No		Yes	No		Yes	No	
Age			.811			<.001			.536			<.001
<66	2179 (47.4%)	2436 (52.6%)		2192 (47.3%)	2438 (52.7%)		786 (17.0%)	3844 (83.0%)		1717 (37.1%)	2913 (62.9%)	
65–73	1158 (47.9%)	1258 (52.1%)		951 (39.4%)	1465 (60.6%)		390 (16.1%)	2026 (83.9%)		1027 (42.5%)	1389 (57.5%)	
>73	1245 (48.1%)	1342 (51.9%)		781 (30.2%)	1806 (69.8%)		417 (16.1%)	2170 (83.9%)		1210 (46.8%)	1377 (53.2%)	
Race			.006			.176			.528			.528
White	3545 (48.3%)	3799 (51.7%)		2989 (40.7%)	4355 (59.3%)		1232 (16.8%)	6112 (83.2%)		2940 (40.0%)	4404 (60.0%)	
Black	543 (43.5%)	705 (56.5%)		543 (43.5%)	705 (56.5%)		197 (15.8%)	1051 (84.2%)		529 (42.4%)	719 (57.6%)	
Other	509 (48.9%)	532 (51.1%)		509 (48.9%)	532 (51.1%)		164 (15.8%)	877 (84.2%)		485 (46.6%)	556 (53.4%)	
Sex			<.001			.139			.235			.018
Male	2468 (50.6%)	2414 (49.4%)		1953 (40.0%)	2929 (60.0%)		829 (17.0%)	4053 (83.0%)		1947 (39.9%)	2935 (60.1%)	
Female	2129 (44.8%)	2622 (55.2%)		1971 (41.5%)	2780 (58.5%)		764 (16.1%)	3987 (83.9%)		2007 (42.2%)	2744 (57.8%)	
Grade			<.001			<.001			<.001			<.001
Well	309 (40.8%)	448 (59.2%)		221 (29.2%)	536 (70.8%)		105 (13.9%)	652 (86.1%)		438 (57.9%)	319 (42.1%)	
Moderate	1585 (49.8%)	1595 (50.2%)		1246 (39.2%)	1934 (60.8%)		416 (13.1%)	2764 (86.9%)		1352 (42.5%)	1828 (57.5%)	
Poorly	2649 (47.4%)	2937 (52.6%)		2409 (43.1%)	3177 (56.9%)		1052 (18.8%)	4534 (81.2%)		2126 (38.1%)	3460 (61.9%)	
Undifferentiated	54 (49.1%)	56 (50.9%)		48 (43.6%)	62 (56.4%)		20 (18.2%)	90 (81.8%)		38 (34.5%)	72 (65.5%)	
Laterality			.834			.117			.328			
Right	2706 (47.6%)	2975 (52.4%)		2277 (40.1%)	3404 (59.9%)		957 (16.8%)	4724 (83.2%)		2321 (40.9%)	3360 (59.1%)	.648
Left	1891 (47.8%)	2061 (52.2%)		1647 (41.7%)	2305 (58.3%)		636 (16.1%)	3316 (83.9%)		1633 (41.3%)	2319 (58.7%)	
Tumor size			.086			<.001			.034			<.001
<38	1783 (47.3%)	1988 (52.7%)		1443 (38.3%)	2329 (61.7%)		578 (15.3%)	3194 (84.7%)		1801 (47.7%)	1971 (52.3%)	
38–58	1371 (46.6%)	1568 (53.4%)		1224 (41.6%)	1715 (58.4%)		514 (17.5%)	2425 (82.5%)		1185 (40.3%)	1754 (59.7%)	
>58	1443 (49.4%)	1479 (50.6%)		1257 (43.0%)	1479 (57.0%)		501 (17.1%)	2421 (82.9%)		968 (33.1%)	1954 (66.9%)	
Primary site			.856			.01			.114			<.001
Upper	2727 (47.5%)	3018 (52.5%)		2371 (41.3%)	3374 (58.7%)		920 (16.0%)	4825 (84.0%)		2277 (39.6%)	3468 (60.4%)	
Middle	196 (47.5%)	217 (52.5%)		167 (40.4%)	246 (59.6%)		79 (19.1%)	334 (80.9%)		178 (43.1%)	235 (56.9%)	
Lower	1290 (48.4%)	1373 (51.6%)		1100 (41.3%)	1563 (58.7%)		442 (16.6%)	2221 (83.4%)		1093 (41.0%)	1570 (59.0%)	
Other	384 (47.3%)	428 (52.7%)		286 (35.2%)	526 (64.8%)		152 (18.7%)	660 (81.3%)		406 (50.0%)	406 (50.0%)	
T stage			<.001			<.001			.201			<.001
T1	559 (52.1%)	513 (47.9%)		480 (44.8%)	592 (55.2%)		162 (15.1%)	910 (84.9%)		167 (15.6%)	905 (84.4%)	
T2	1298 (49.9%)	1302 (50.1%)		1265 (48.7%)	1335 (51.3%)		408 (15.7%)	2192 (84.3%)		492 (18.9%)	2108 (81.1%)	
T3	1178 (46.4%)	1363 (53.6%)		987 (38.8%)	1554 (61.2%)		438 (17.2%)	2103 (82.8%)		1214 (47.8%)	1327 (52.2%)	
T4	1562 (45.7%)	1858 (54.3%)		1192 (34.9%)	2228 (65.1%)		585 (17.1%)	2835 (82.9%)		2081 (60.8%)	1339 (39.2%)	
N stage			<.001			<.001			<.001			<.001
N0	959 (38.7%)	1516 (61.3%)		1025 (41.4%)	1450 (58.6%)		298 (12.0%)	2177 (88.0%)		946 (38.2%)	1529 (61.8%)	
N1	395 (46.4%)	456 (53.6%)		387 (45.5%)	464 (54.5%)		127 (14.9%)	724 (85.1%)		269 (31.6%)	582 (68.4%)	
N2	2244 (50.8%)	2177 (49.2%)		1811 (41.0%)	2601 (59.0%)		790 (17.9%)	3631 (82.1%)		1763 (39.9%)	2658 (60.1%)	
N3	999 (53.0%)	887 (47.0%)		701 (37.2%)	1185 (62.8%)		378 (20.0%)	1508 (80.0%)		976 (51.7%)	910 (48.3%)	
Surgery			<.001			.185			<.001			.882
Yes	184 (23.9%)	586 (76.1%)		331 (43.0%)	439 (57.0%)		54 (7.0%)	716 (93.0%)		318 (41.3%)	452 (58.7%)	
No	4413 (49.8%)	4450 (59.2%)		3593 (40.5%)	5270 (59.5%)		1539 (17.4%)	7324 (82.6%)		3636 (41.0%)	5227 (59.0%)	
Radiation			.051			<.001			<.001			<.001
Yes	2504 (48.6%)	2643 (51.4%)		3141 (61.0%)	2006 (39.0%)		689 (13.4%)	4458 (86.6%)		1549 (30.1%)	3598 (69.9%)	
No	2093 (46.7%)	2393 (53.3%)		783 (17.5%)	3703 (82.5%)		904 (20.2%)	3582 (79.8%)		2405 (53.6%)	2081 (46.4%)	
Chemotherapy			<.001			.264			.033			.381
Yes	2909 (49.2%)	2998 (50.8%)		2308 (40.3%)	3527 (59.7%)		939 (15.9%)	4968 (84.1%)		2404 (40.7%)	3503 (59.3%)	
No	1688 (45.3%)	2308 (54.7%)		1544 (41.4%)	2182 (58.6%)		654 (17.6%)	3072 (82.4%)		1550 (41.6%)	2176 (58.4%)	

The metastatic patterns of organ metastatic lung AD were shown in Table 2. In all, there were 15 possible metastatic patterns. These modes include 4 single metastases, 6 two sites metastases, 4 three sites metastases, and 1 four sites metastases. In patients with a single metastasis, analysis of data represented that the brain (21.20%) was the most common site for organ metastatic lung AD, followed by bone (21.16%), lung (18.99%), and liver (3.50%) metastasis. For 2 sites metastases, the proportion of bone and lung metastasis was 7.53% at most. The proportion of brain and liver metastases (1.16%) was relatively small.

**Table 2:** Frequencies of combination metastasis.

	Distant metastatic lung adenocarcinoma	
Features	Number	Percentage (%)
One site		
Only bone	2038	21.16
Only brain	2042	21.20
Only liver	337	3.50
Only lung	1829	18.99
Two sites		
Bone and brain	608	6.31
Bone and liver	377	3.91
Bone and lung	725	7.53
Brain and liver	112	1.16
Brain and lung	498	5.17
Liver and lung	152	1.58
Three sites		
Bone and brain and liver	165	1.71
Bone and brain and lung	300	3.11
Bone and liver and lung	251	2.61
Brain and liver and lung	66	0.69
Four sites		
Bone and brain and liver and lung	133	1.38

### 3.3. Survival analyses of patients with 4 single metastases

**Figure 1.**
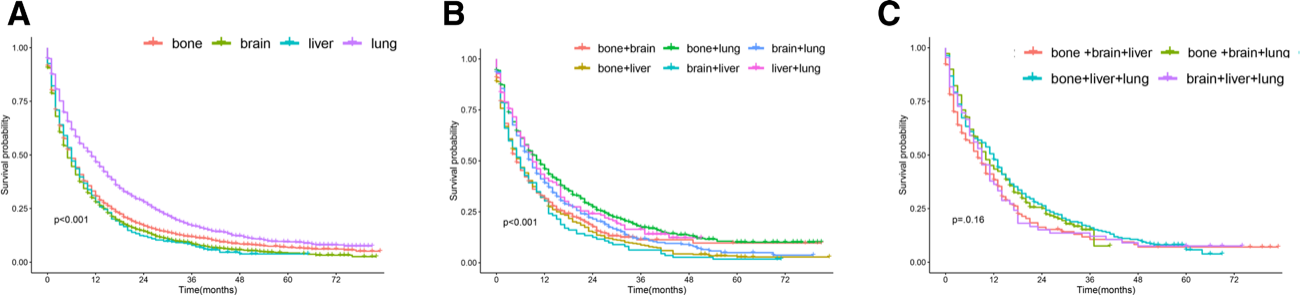
(A) Kaplan–Meier curves and Log-rank test for overall survival according to 4 single metastases. (B) Kaplan–Meier curves and Log-rank test for overall survival according to 2 metastatic sites. (C) Kaplan–Meier curves and Log-rank test for overall survival according to 3 metastatic sites.

In addition, just as shown in Table 3, this study performed univariate survival analyses to estimate the effect of single metastases on overall survival (OS). We found that metastasis site, T stage, tumor size and radiation had significant effect on OS. Lung metastasis matched with the other single metastases exhibited relatively good OS, whose mean of survival was 15.337 months (95% CI 1.332,1.698 *P* < .001). Regrettably, the mean survival time of those who suffered brain metastasis was 10.006 months (95% CI 1.267,1.454 *P* < .001). Next, we brought significant factors into the multivariate survival analysis. Table 4 showed that metastasis site and tumor size were independent prognostic factors (*P* < .001). As shown in Figure 1A, the median survival times of patients with single lung metastasis (11 months; 95% CI 0.470–0.516) was better than brain metastasis (5 months; 95% CI 0.465–0.508), bone metastasis (5 months; 95% CI 0.504–0.547) and liver metastasis (6 months; 95% CI 0.483–0.590). In addition, the long-term survival benefit of patients with single lung metastasis was better than that of patients with single brain metastasis, bone metastasis and liver metastasis. Long-term survival benefits of brain metastases, bone metastases and liver metastases were similar.

**Table 3:** Univariate survival analysis of patients with 4 single metastases.

Risk factors	Mean of survival months	95% CI	*P* value
Metastasis site			
Bone metastasis	11.122		<.001
Brain metastasis	10.006	(1.267, 1.454)	<.001
Liver metastasis	10.586	(1.414, 1.622)	<.001
Lung metastasis	15.337	(1.332, 1.698)	<.001
Age			
<66	11.778		.303
65–73	11.895	(0.984, 1.118)	.141
>73	12.324	(0.972, 1.126)	.230
Race			
White	11.905		.791
Black	12.112	(0.943, 1.129)	.500
Other	12.170	(0.915, 1.143)	.691
Sex			
Male	11.672		
Female	12.246	(0.986, 1.097)	.146
Grade			
Well	11.781		.626
Moderate	11.978	(0.761, 1.269)	.893
Poorly	11.945	(0.815, 1.323)	.761
Undifferentiated	13.395	(0.825, 1.334)	.694
Laterality			
Right	11.897		
Left	12.052	(0.956, 1.066)	.736
Tumor size			
<38	14.307		.001
38–58	11.260	(0.674, 0.767)	.001
>58	9.737	(0.822, 0.940)	.001
Primary site			
Upper	11.742		.242
Middle	11.121	(0.974, 1.191)	.148
Lower	12.481	(0.923, 1.272)	.328
Other	12.255	(0.919, 1.139)	.677
T stage			
T1	11.041		.003
T2	11.360	(1.055, 1.256)	.002
T3	12.226	(1.036, 1.187)	.003
T4	12.714	(0.971, 1.120)	.249
N stage			
N1	11.783		.206
N2	11.714	(0.996, 1.174)	.061
N3	11.947	(0.983, 1.221)	.099
N4	12.451	(0.997, 1.164)	.059
Surgery			
Yes	11.437	(0.866, 1.026)	.175
No	12.022		
Radiation			
Yes	10.950		
No	12.967	(0.811, 0.902)	<.001
Chemotherapy			
Yes	11.854		
No	12.128	(0.918, 1.024)	.262

**Table 4:** Multivariate survival analysis of patients with 4 single metastases.

Risk factors	Hazard ratio (HR)	95% CI	*P* value
Metastasis site			
Bone metastasis			<.001
Brain metastasis	1.316	(1.223, 1.417)	<.001
Liver metastasis	1.459	(1.346, 1.581)	<.001
Lung metastasis	1.440	(1.274, 1.628)	<.001
T stage			
T1			.882
T2	1.017	(0.929, 1.112)	.716
T3	0.983	(0.916, 1.055)	.635
T4	1.003	(0.933, 1.077)	.945
Tumor size			
<38			<.001
38–58	0.748	(0.701, 0.798)	<.001
>58	0.895	(0.837, 0.958)	<.001
Radiation			
Yes			
No	1.024	(0.963, 1.088)	.447

### 3.4. Survival analyses of patients with different combinations of metastases

There were 6 types of patients who suffered 2 metastatic sites. The proportion of bone and lung metastasis was 7.53% at most. Table S1, Supplemental Digital Content 1, http://links.lww.com/MD/I770 presented that age, race, sex, grade, laterality, primary site, N stage, surgery, radiation and chemotherapy were not bound up with the prognostic of patients in the univariate survival analysis (all, *P* > .05). Data analysis suggested that metastatic pattern, tumor size and T stage were related to survival. Figure 1B presents the survival curves of patients with 2 metastatic sites. The median survival times of patients with bone and lung metastasis (10 months; 95% CI 0.469–0.542) was better than bone and brain metastasis (5 months; 95% CI 0.454–0.533), bone and liver metastasis (5 months; 95% CI 0.462–0.564), brain and liver metastasis (5 months; 95% CI 0.433–0.619), brain and lung metastasis (8 months; 95% CI 0.463–0.551) and liver and lung metastasis (9 months; 95% CI 0.421–0.582).

Similarly, we went deep into the situations of patients with 3 metastatic sites. There were 4 metastatic patterns, including bone and brain and liver metastasis, bone and brain and lung metastasis, bone and liver and lung metastasis, brain and liver and lung metastasis. As shown in Table S2, Supplemental Digital Content 2, http://links.lww.com/MD/I771, univariate survival analysis results presented that metastatic pattern, age, race, sex, grade, laterality, primary site, N stage, surgery, radiation and chemotherapy had no effect on the OS (all, *P* > .05). Tumor size and T stage were still related to survival. Figure 1C presents the survival curve of the 4 types of metastatic tumors. We found that metastatic pattern had no effect on the OS (*P* > .05).

### 3.5. Internal verification

**Figure 2.**
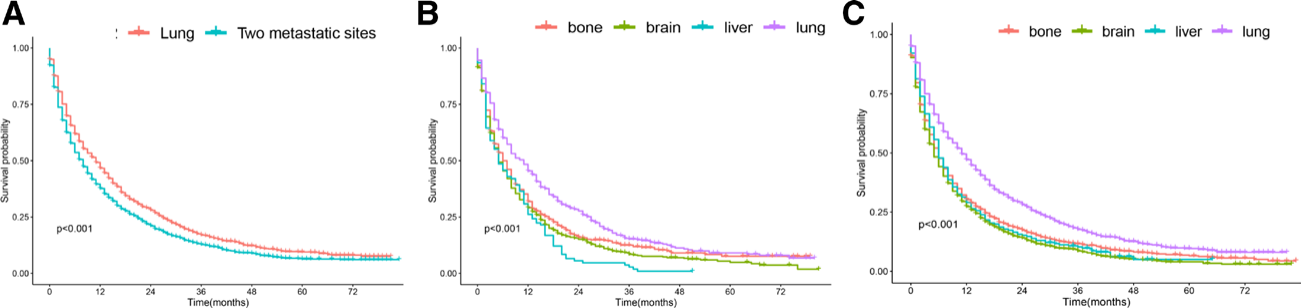
(A) Kaplan–Meier curves and Log-rank test for overall survival according to lung metastatic and 2 metastatic sites. (B) Kaplan–Meier curves and Log-rank test for overall survival based on training cohort. (C) Kaplan–Meier curves and Log-rank test for overall survival based on validation cohort.

As shown in Figure 2A, it was found through comparison that the median survival time of patients with single lung metastasis (11 months, 95% CI 0.470, 0.516) are longer than that of patients who suffered 2 metastatic sites (7 months, 95% CI 0.486, 0.525).

In order to further verify the result, we randomly divided all patients into training (n = 4374) and validation (n = 1872) cohorts at the ratio of 7:3. And as shown in Figure 2B, in the experimental group, lung metastasis matched with the other single metastases exhibited relatively good OS, whose mean of survival was 11 months (95% CI 0.442, 0.529 *P* < .001). And as shown in Figure 2C, in the control group, lung metastasis matched with the other single metastases exhibited relatively good OS, whose mean of survival was 11 months (95% CI 0.469, 0.523 *P* < .001).

## 4. Discussion

This study proved that single lung metastasis represented relatively good OS. Metastatic pattern was an independent prognostic element in organ metastatic lung AD patients. For patients with 3 metastatic sites, data analysis suggested that metastatic pattern had no effect on OS (*P* > .05).

Past studies had made known that metastases of lung AD were related to the expression of certain genes, including anillin actin-binding protein (ANLN), ubiquitin-conjugating enzyme E2S (UBE2S), brahma-related gene 1 (BRG1), ras homolog family member V (RHOV), transcription factor AP-2α (TFAP2A), mi-RNA-34, and at-rich interaction domain 1A (ARID1A).^[13–18]^ However, these studies were all ignorant of the prognostic impact of metastatic pattern. Therefore, we conducted a deep analysis on the patterns of metastasis to better understand the prognosis of organ metastatic lung AD. These results may be helpful for medical workers to assess patients’ conditions, formulate treatment plans and provide practical help.

In our research, we discovered that the most common site of organ metastatic lung AD was the brain, and the least common site of metastasis was the liver. Similarly, previous articles have shown that the most common sites of metastasis in lung AD are brain and bone (25%–40%).^[12,19,20]^ On the basis of demographics and clinical features of patients, we found that older patients (>73) were more likely to have brain metastasis and lung metastasis, black people were more related to bone metastasis than white people, and compared with men, women were more prone to bone and lung metastasis. Additionally, patients with relatively large masses (>58 mm), positive lymph nodes and low histological grade were more likely to have organ metastasis.

Finally, we explored the influence of metastasis patterns on the survival of lung AD. The median survival time of single lung metastasis patients was 11 months (95% CI 0.470–0.516), showing a relatively better prognosis than those with liver, brain and bone metastases. Similarly, Ren reported that the OS of single brain metastasis and single bone metastasis is relatively good, while the single liver metastasis is relatively poor.^[21]^

For the 2 sites metastasis group, our data showed that the median survival times of bone and lung metastasis patients (10 months; 95% CI 0.469–0.542) were relatively good. For the 3 sites metastasis group, our data showed that metastatic pattern, age, race, sex, grade, laterality, primary site, N stage, surgery, radiation, and chemotherapy had no effect on the OS (all, *P* > .05). Tumor size and T stage were still proven related to survival, while metastatic pattern had no effect on the OS. This suggests that the prognosis of these patients depends largely on the metastases themselves.

## Author contributions

**Conceptualization:** Jin Wang.

**Data curation:** Yong Wang, Chenchen Wang, Fang Wang, Simeng Chen.

**Formal analysis:** Xaiomin Li.

**Project administration:** Baobao Fu, Yanshun Zhang.

**Software:** Jin Wang, Qianqian Jia.

**Writing – original draft:** Xiaowei Tie.

## Supplementary Material
