# Supplementary material for: The prognostic effect of metastasis patterns on overall survival in organ metastatic lung adenocarcinoma
Source: Medicine (Baltimore). 2022 Apr 7;102(14):e33297. doi: 10.1097/MD.0000000000033297 (PMC10082283; doi:10.1097/MD.0000000000033297)
Supplement: Supplementary file 1 [file medi-102-e33297-s001.pdf]

Table S1 Univariate survival analysis of patients with two metastatic sites metastases

Risk factors	Mean of survival months	95% CI	*p*
**Metastasis site**			
Bone and liver metastasis	11.387		<0.001
Bone and lung metastasis	15.284	(0.924,1.124)	0.407
Brain and liver metastasis	10.723	(0.626,0.798)	<0.001
Brain and lung metastasis	12.076	(0.914,1.383)	0.267
Liver and lung metastasis	14.493	(0.736,0.957)	0.009
Bone and brain metastasis	9.416	(0.627,0.925)	0.006
**Age**			
<66	11.691		0.030
65 to 73	12.359	(1.037,1.282)	0.008
>73	13.572	(0.961,1.223)	1.191
**Race**			
White	12.351		0.552
Black	11.589	(0.914,1.195)	0.516
Other	13.061	(0.928,1.320)	0.257
**Sex**			
Male	12.451		
Female	12.229	（0.920,1.092）	0.955
**Grade**			
Well	11.491		0.246
Moderate	12.996	(0,505,1.345)	0.438
Poorly	12.127	(0.459,1.172)	0.195
Undifferentiated	9.100	(0.496,1.258)	0.320
**Laterality**			
Right	11.908		
Left	12.975	(0.845,1.007)	0.07
**Tumor size**			
<38	14.770		<0.001
38 to 58	11.869	(0.591,0.728)	<0.001
>58	9.718	(0.756,0.938)	<0.001
**Primary Site**			
Upper	12.506		0.749
Middle	11.327	（0.905,1.249）	0.454
Lower	11.898	(0.859,1.442)	0.416
Other	13.204	(0.921,1.297)	0.309
**T stage**			
T1	9.665		0.001

T2	11.248	(1.134,1.562)	0.000
T3	12.698	(1.054,1.306)	0.006
T4	13.274	(0.967,1.196)	0.180
**N stage**			
N1	12.406		0.980
N2	12.866	(0.851,1.110)	0.676
N3	12.323	(0.835,1.186)	0.960
N4	12.158	(0.886,1.100)	0.813
**Surgery**			
Yes	12.371		
No	11.688	(0.789,1.255)	0.966
**Radiation**			
Yes	11.871		
No	12.989	(0.871,1.036)	0.244
**Chemotherapy**			
Yes	12.518		
No	12.067	(0.958,1.142)	0.314
