# Supplementary material for: The prognostic effect of metastasis patterns on overall survival in organ metastatic lung adenocarcinoma
Source: Medicine (Baltimore). 2022 Apr 7;102(14):e33297. doi: 10.1097/MD.0000000000033297 (PMC10082283; doi:10.1097/MD.0000000000033297)
Supplement: Supplementary file 2 [file medi-102-e33297-s002.pdf]

Table S2 Univariate survival analysis of patients with three metastatic sites metastases

Risk factors	Mean of survival months	95% CI	*p*
**Metastasis site**			
Bone and brain and liver metastasis	11.260		0.188
Bone and brain and lung metastasis	12.440	(0.788,1.449)	0.667
Bone and liver and lung metastasis	16.910	(0.656,1.159)	0.345
brain and liver and lung metastasis	14.860	(0.654,1.151)	0.868
**Age**			
<66	13.230		0.610
65 to 73	14.290	(0.908,1.333)	0.330
>73	14.680	(0.840,1.312)	0.670
**Race**			
White	14.250		0.117
Black	11.270	(0.771,1.222)	0.801
Other	13.920	(0.916,1.667)	0.166
**Sex**			
Male	14.120		
Female	13.520	（0.846,1.150）	0.859
**Grade**			
Well	14.544		0.661
Moderate	13.234	(0.424,1.601)	0.568
Poorly	14.042	(0.514,1.736)	0.855
Undifferentiated	13.750	(0.548,1.817)	0.995
**Laterality**			
Right	13.030		
Left	14.870	(0.759,1.034)	0.125
**Tumor size**			
<38	15.780		<0.001
38 to 58	13.050	(0.576,0.838)	<0.001
>58	11.420	(0.725,1.085)	0.244
**Primary Site**			
Upper	14.070		0.744
Middle	10.560	（0.812,1.325）	0.772
Lower	13.460	(0.811,1.951)	0.305
Other	14.780	(0.775,1.315)	0.946
**T stage**			
T1	12.610		0.021
T2	10.500	(0.972,1.993)	0.071
T3	13.480	(1.089,1.691)	0.007

T4	15.260	(0.969,1.382)	0.106
**N stage**			
N1	12.830		0.630
N2	15.730	(0.721,1.178)	0.515
N3	14.390	(0.603,1.164)	0.290
N4	12.960	(0.755,1.082)	0.273
**Surgery**			
Yes	13.790		
No	17.000	(0.518,1.931)	0.999
**Radiation**			
Yes	12.740		
No	15.760	(0.756,1.039)	0.136
**Chemotherapy**			
Yes	13.445		
No	14.397	(0.796,1.087)	0.363
